# A cholinergic hypothesis of the unconscious in affective disorders

**DOI:** 10.3389/fnins.2013.00220

**Published:** 2013-11-22

**Authors:** Costa Vakalopoulos

**Affiliations:** Private PracticeMelbourne, VIC, Australia

**Keywords:** muscarinic receptor, serotonin, memory, hippocampus

## Abstract

The interactions between distinct pharmacological systems are proposed as a key dynamic in the formation of unconscious memories underlying rumination and mood disorder, but also reflect the plastic capacity of neural networks that can aid recovery. An inverse and reciprocal relationship is postulated between cholinergic and monoaminergic receptor subtypes. M1-type muscarinic receptor transduction facilitates encoding of unconscious, prepotent behavioral repertoires at the core of affective disorders and ADHD. Behavioral adaptation to new contingencies is mediated by the classic prototype receptor: 5-HT1A (Gi/o) and its modulation of M1-plasticity. Reversal of learning is dependent on increased phasic activation of midbrain monoaminergic nuclei and is a function of hippocampal theta. Acquired hippocampal dysfunction due to abnormal activation of the hypothalamic-pituitary-adrenal (HPA) axis predicts deficits in hippocampal-dependent memory and executive function and further impairments to cognitive inhibition. Encoding of explicit memories is mediated by Gq/11 and Gs signaling of monoamines only. A role is proposed for the phasic activation of the basal forebrain cholinergic nucleus by cortical projections from the complex consisting of the insula and claustrum. Although controversial, recent studies suggest a common ontogenetic origin of the two structures and a functional coupling. Lesions of the region result in loss of motivational behavior and familiarity based judgements. A major hypothesis of the paper is that these lost faculties result indirectly, from reduced cholinergic tone.

## Introduction

“The consistent impairments in memory function… suggest that as we learn more about memory mechanisms in humans we shall learn more about depression” (Austin et al., 2001).

### Some preliminaries

A satisfactory biological theory of conscious and unconscious mental phenomena that can unite psychological and neuroscientific bodies of work into a cogent theory, a proper science of the mind, remains elusive. The present paper focuses on specific neuromodulatory roles that underlie various aspects of cognition in a dual process theory of mind. The organizational principles of parallel conscious and unconscious cognitive structures were presented within anatomical and pharmacological frameworks (Vakalopoulos, 2005a,b, 2006). The derivation of this network theory is from an all-unifying process of motor efference copy where it is proposed that the foundations of cognition and learning are inscribed by reentrant motor algorithms sourced from the organism's direct interaction with the environment. The emergence of conscious and unconscious neural properties of neural networks is synonymous with encoding through reentry of discovered motor algorithms by the organism that categorize sensory input at a pre-phenomenological stage. That is the organism attaches meaning to the input through the discovery of successful motor algorithms. Thus, phenomenology as an emergent property of neural networks in turn is a prediction of all possible behavioral application to sensory data. The differentiation between each of the two cognitive forms (conscious and unconscious) is determined by the specific nature of the reentrant algorithm defined by simple (unconscious) and complex (conscious) premotor networks (Vakalopoulos, 2005a).

A dual process theory of cognition not only suggests segregated neural networks, but also a distinct pharmacology (Vakalopoulos, 2006). A balanced antagonism and inverse relationship between monoaminergic and cholinergic receptor subtypes is proposed in explaining the relationship between cognitive inhibition, memory and mood dysregulation. According to this hypothesis, monoaminergic receptors facilitate conscious and inhibit unconscious networks and permit reversal of learning by triggering plastic changes in synaptic couplings. In contrast, muscarinic cholinergic receptors have an inverse and symmetrical relationship, i.e., facilitate unconscious and inhibit conscious networks. This setup is inferred from general properties and relative distributions of the various receptor subtypes. Reversal of learning is mediated by the convergence of serotonergic 5-HT1A-M1 muscarinic receptor signaling and is the putative point of action of various cognitive and pharmacological therapies for mood disorder (Vakalopoulos, 2007). Reversal of learning is a common experimental animal paradigm for cognitive flexibility (impaired in depression) when contingency factors change, altering requirements of behavior for acquiring rewards. It is believed to rely on effective monoaminergic signaling.

### What the paper purports to achieve, in summary:

- Monoaminergic modulation of cholinergic facilitated networks creates a novel dimension to the monoaminergic deficit view of affective disorders. Normal interaction is mediated through receptor subtypes and alters prepotent, but maladaptive behavior and is the basis of rewiring cortical networks.
- Monoamine phasic increases encode concurrent cortical traces as explicit memories. It is mediated by the theta rhythm of the hippocampus and its projections that relay to monoaminergic midbrain and pontine nuclei. The proposal of this simplified mechanism embraces and unifies disparate findings of affective disorders including explicit memory impairment and poor cognitive flexibility. The model directly links the increases in cortisol levels and hippocampal dysfunction to reduced monoamine levels.
- Muscarinic cholinergic phasic activation, mediated by distinct cortical centers to those that activate monoaminergic nuclei is proposed to directly encode implicit cortical traces. Much affective discourse i.e., rumination, negative thinking is triggered by cues to implicit motivational memories and thus, explains its prepotent nature. It also provides a basis for the dissociation of explicit memory loss from preserved implicit memory, a feature also typical of major depressive illness. There is evidence that cholinergic activation itself increases cortisol response. The proposal is that part of the mechanism of selective serotonin reuptake inhibitors (SSRIs) is switching off the HPA axis via its antagonism of cholinergic input.
- Finally, the paper extends the cholinergic hypothesis to an affective spectrum of disorders. A number of features, such as genetic vulnerability factors, high degree of overlapping comorbidity, cortisol impaired hippocampal function as a result of trauma, cognitive deficits and response to SSRIs are shared by the spectrum of mood and anxiety disorders

## The insula cortex and cholinergic activation

If the hippocampal theta rhythm is associated with increased activity within monoaminergic projection neurons and is the putative basis for encoding of declarative memories within the neocortex, what is the evidence for limbic or paralimbic activation of the cholinergic basal forebrain? Is there a physiological index of implicit affective processes, including the formation of prepotent behavioral repertoires that are predicated on muscarinic signaling?

There are some limited tracing studies looking at afferent cortical input to the basal forebrain and the nucleus Basalis of Meynert, in particular. One of the most prominent inputs is the anterior agranular insula (Russchen et al., 1985; Grove, 1988). Lesions of the insula can produce effects that are as dramatic as those of the hippocampus. For example, a rat's incentive memory in a satiety task is attenuated during the extinction phase (Balleine and Dickinson, 2000). Profound disruption of cigarette addiction is a recent finding of insula lesions (Naqvi et al., 2007). A more general role in drug addiction is suggested by a study of cue-related drug craving. Amphetamine-treated rats showed inverse place preference with reversible inactivation of the insula (Contreras et al., 2007). Place conditioning to amphetamine is paralleled by an increase in the immediate early gene Fos immunoreactivity of the insula in addition to lateral hypothalamic orexin neurons. Orexin neurons project to the basal forebrain and increase firing of cholinergic corticopetal cells (Fadel and Burk, 2010).

A commonly accepted view of the limbic system and the insula, in particular, in conscious self-awareness has been questioned recently by a rare case of viral encephalitic obliteration of the entire limbic system of both hemispheres (Philippi et al., 2012). Roger suffered damage to medial temporal areas associated with anterograde memory loss, but also extended to bilateral insula and basal forebrain. Paradoxically he is happier now than prior to his illness with no evidence for depressive symptoms. His neuropsychological profile led the investigators to conclude that the limbic system is more closely allied to memory function than it does with emotional expression (Feinstein et al., 2010). However, implicit memory and emotional dysregulation are inextricably linked and the cholinergic basal forebrain provides a critical element of neuromodulation (Vakalopoulos, 2006).

Recognition tasks can index implicit memory in the contexts of declarative or explicit memory deficit (Vakalopoulos, 2006). An analogy is represented by the dichotomy between explicit recall and familiarity-based judgments. Roger demonstrates as severe an impairment in visual recognition for faces and words and delayed recognition component of the Rey Auditory Verbal Learning Test as for delayed recall performance. This is in striking contrast to a series of cases of developmental damage to the hippocampus where a clear dissociation exists between recall and recognition (Vargha-Khadem et al., 1997). Word recognition is intact in a separate case of focal bilateral hippocampal damage secondary to meningitis, which spared the perirhinal cortex (Aggleton et al., 2005). Word recognition was actually slightly superior to that of controls while recall was severely impaired. The case of Roger supports the contention that the additional loss is learning of some forms of implicit memory is a consequence of damage extending to critical areas such as the insula and basal forebrain.

The idea that the perirhinal cortex is involved in familiarity-based judgments has gained considerable favor. Selective surgical resection of the perirhinal and entorhinal cortices, sparing the hippocampus, revealed impaired word recognition, but preserved recollection (Bowles et al., 2007). Noteworthy, was a suboptimal performance on semantic fluency. A double dissociation was confirmed in another series of surgical patients with impaired recollection, but overall intact recognition (Bowles et al., 2010). These patients had selective unilateral resection of the hippocampus and amygdala, but spared the perirhinal cortex. Like the insula, the perirhinal and entorhinal cortex both project to the basal forebrain (Grove, 1988). Involvement of the temporopolar cortex is a potential source of disruption of frontotemporal white matter tracts. There is also a rich projection from the amygdala to the basal forebrain. The differential contributions to activation of distributed cholinergic networks and precise roles in familiarity based cognitive performance remain open questions.

### Claustrum: the “non-declarative” hippocampus

The claustrum is a thin sheet of gray matter wedged between deep layers of the insula cortex and the putamen and separated from these structures by the white matter tracts of the extreme and external capsules, respectively. A high definition diffusion tractography study confirms widespread cortical connections (Park et al., 2012), but it's functional status and it's relation to the insula remain controversial if unknown. This is an enigmatic structure of some importance (Crick and Koch, 2005).

**Figure 1:**
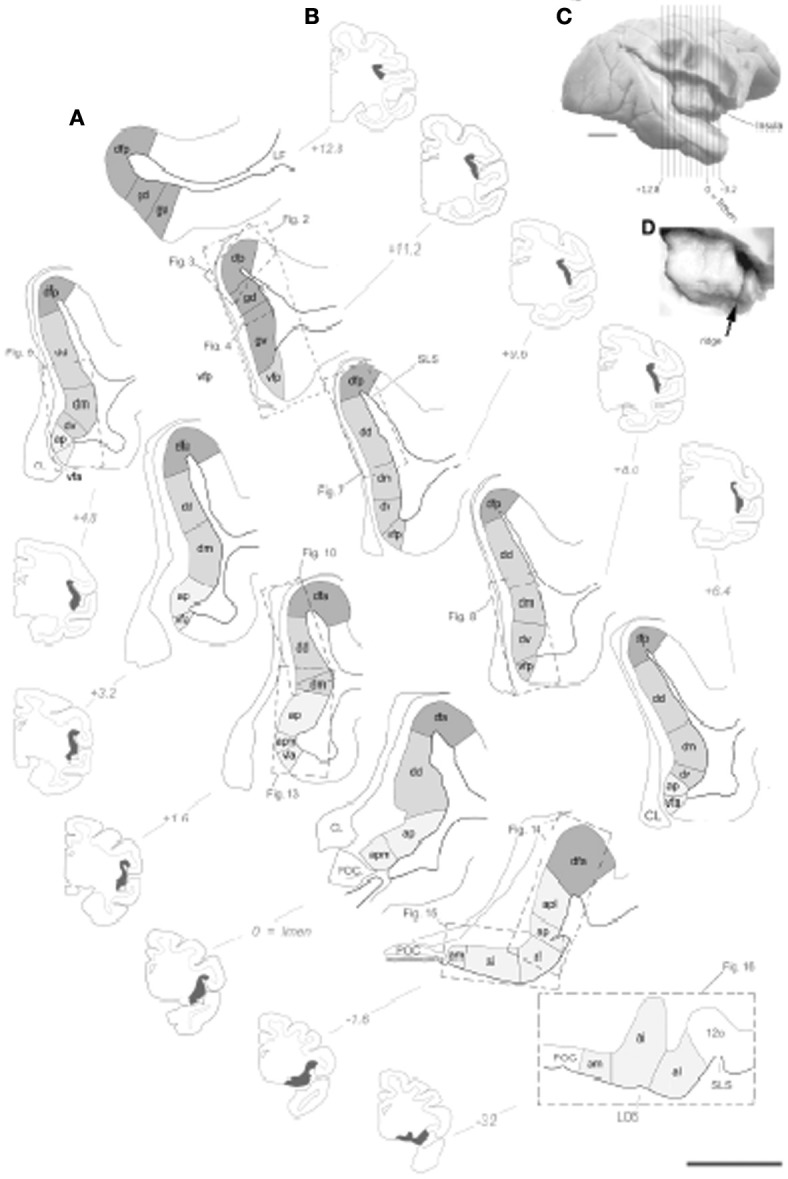
**Illustration of the topographical localization of the 15 distinct architectonic areas of the insular cortex in one set of drawing of 11 coronal sections from the right hemisphere from one representative case cm6R. (A)** Drawing of the 15 architectonic areas and borders. The granular dysgranular and agranular areas are distinguished by darker, intermediate and light gray tones, respectively. Scale bar = 2.5 mm. **(B)** Low magnification and simplified drawing of the entire coronal section from which the drawings in panel **(C)** were made. The extent of insular cortex shown in panel **(C)** is represented by the darkened areas. The anteroposterior position of the section is indicated on the right or left of the section using the limen insulae as the zero. **(C)** Approximate anteroposterior position of the coronal section juxtaposed on a lateral view photograph of a different brain in which the insula was exposed by dissection.**(D)** Higher magnification of the same lateral view showing the anterior vertical ridge of the insula (arrow).

One study did not show uptake by the claustrum with a large deposit of retrograde tracer in the insula (Smith and Alloway, 2010). This contradicts earlier studies showing extensive connections between the two structures in the rat and cat (Witter et al., 1988; Behan and Haberly, 1999). Morphologically, early anatomists considered the claustrum part of the deep layer VI of the insula or as a clear marker that defined the extent of the insular cortex (see discussion in Nieuwenhuys, 2012). In a recent architectonic analysis the insula cortex is coextensive with the claustrum along almost its entire anterior-posterior and dorsal-ventral extent (Figure 1) (Evrard et al., 2013). Clear separation of the claustrum emerges only later in the phylogenetic history of the primate (Park et al., 2012).

**Figure 2:**
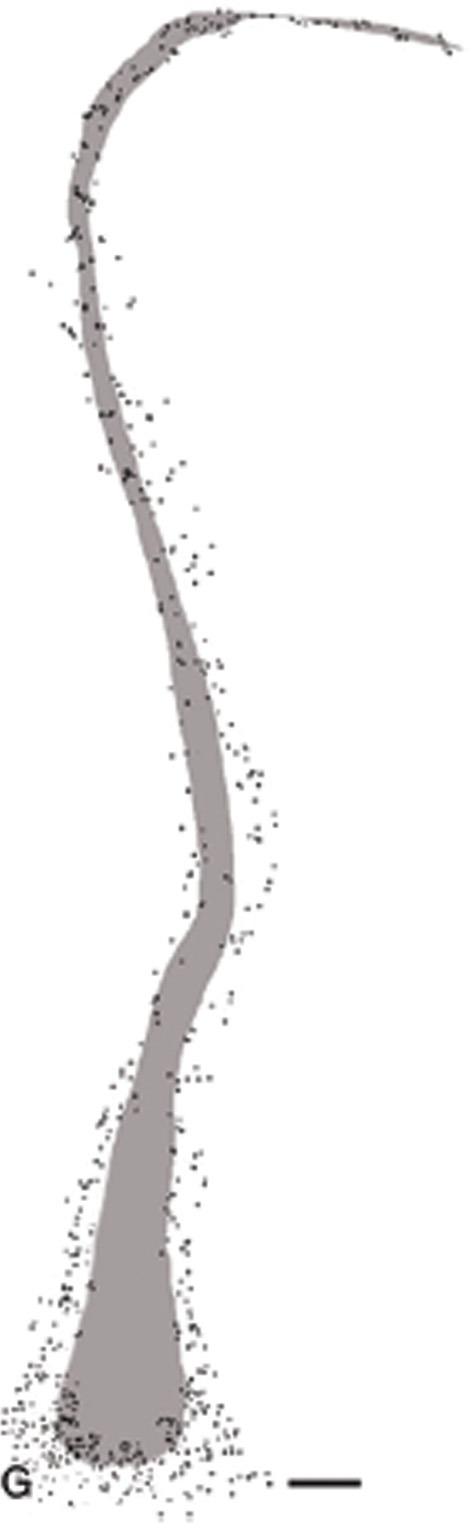
**Shaded area represents the calustrum as defined by parvalbumin immunoraectivity**. The surrounding and embedded dots are immunoreactive deep layer VI cells of the insula cortex. Permission to reprint from Oxford University Press, (Mathur et al., 2009), this figure is not included under Frontiers' CC-BY license policy.

The claustrum being a thin sheet of neural tissue and its close proximity to the insula make it difficult to interpret the likely spread of the injection sites. However, proteomic analysis does suggest a common ontogeny and connectivity (Miyashita et al., 2005; Mathur et al., 2009; Pirone et al., 2012). A robust functional coupling is suggested by the demonstration of the claustrum embedded in layer VI of the insula cortex, which separates it from the adjacent white matter bundles (Figure 2). The anatomical associations lend plausibility to the proposal that subortical projections of the claustrum are mediated in part by deep insula layers. Phasic activation of cholinergic corticopetal projections is one such function of immense putative significance for facilitating widespread encoding of cortical traces that represent implicit memories of events and learning of procedural skills.

The network proposed to subserve the basis for preserved, but subconscious cognitive performance in blindsight has been partly validated (Vakalopoulos, 2008; Schmid et al., 2010). Evidence for linking the claustrum to this network is longstanding and comes from a study of the retrograde tracer HRP (horseradish peroxidase) limited to the superficial layers of striate cortex in lower primate species (Carey et al., 1980). Although, specificity is difficult to achieve with such deposits, projections to this region include a network comprised of the claustrum, koniocellular layers of the lateral geniculate nucleus and the intralaminar thalamus. The findings thus support an association between cortical and subcortical nodes of the cognitive subconscious as originally proposed (Vakalopoulos, 2005a,b) with the nexus of information flow to the claustrum, the “non-declarative hippocampus.”

Encoding of a trace would observe a modified Hebbian rule of concurrently active cortical networks and phasic cholinergic neuromodulation of synaptic strengths. The latter is mediated by the unique properties of neurons within the claustrum with an emergent and enhance synchronized output. Activity in the claustrum is set up in turn by the projection of perceptual information (presumably unconscious), in other words a self-selecting mechanism. Convergence of signaling recruits glutamatergic receptors including those of NMDA and the motivational modulation mediated by mesolimbic dopamine systems. For example D3 dopamine receptor does interact with NMDAR and increases Akt phophorylation (Salles et al., 2013; Sokoloff et al., 2013). The advent of a system of concurrent signaling ensures a degree of specificity and malleability.

A number of experimental properties support the view of the claustrum as a generator of neuromodulator activity and further studies have shown involvement in non-declarative or familiarity-based judgments. The limbic region surrounding the piriform cortex has long been regarded as one of the most epileptogenic (Tseng and Haberly, 1989). Very low seizure thresholds in the deep agranular insula and perirhinal cortex and in particular, the ventral claustrum are considered the sites of origin of epileptiform discharge (Hoffman and Haberly, 1996; Demir et al., 1998). This evidence hints at a unique feature in the coupling of neurons leading to synchronized bursting and the putative phasic responses of the cholinergic basal forebrain. It is also consistent with a functional coupling of the claustrum to the insula. Conversely, the proposed generalized property of the paralimbic mantle from insula and claustrum to the amygdala hippocampal complex in activating cholinergic and monoaminergic cell groups is the basis of its kindling potential.

Implicit memories including semantic knowledge are implicated in a number of experimental paradigms involving recognition, fluency, intuition, and skill learning. Performance in all of these tasks has been associated with claustrum activation on fMRI (Volz and von Cramon, 2006; Volz et al., 2010; Baugh et al., 2011; Tian et al., 2011). In a visuomotor adaptation task increased activity in the claustrum only became significant in later trials reflecting successful acquisition of a new skill (Baugh et al., 2011). Reversible bilateral lesions of the claustrum were documented in a 12 year old girl with status epilepticus (Sperner et al., 1996). The clinical picture resulted in a severe encephalopathy with psychotic features resembling an anticholinergic delirium. Improvement in symptoms followed resolution of MRI findings of localized oedema.

## Evidence for a cholinergic theory of affective disorders

Muscarinic, like monoamine, receptors are coupled to G-proteins and are similarly, ubiquitously distributed. Anticholinergic agents can induce euphoria and cause memory deficits. An inverse functional relationship exists between monoamine and muscarinic receptors, which is well characterized for dopamine D1-M4, D2-M1Rs in the basal ganglia. Anticholinesterases, which increase ACh (acetylcholine) levels induce dysphoric symptoms, particularly in those so predisposed (Vakalopoulos, 2007) and reverse the effects of antidepressant treatment. A monoaminergic-cholinergic imbalance of sleep disturbance in major depression is suggestive of a cholinergic hypersensitivity model of the disorder (McCarley, 1982; Sitaram et al., 1982; Berger and Rieman, 1993; Buysse et al., 2006). Increased REM (rapid eye movement) sleep and shorter latency to REM is strongly associated with clinical depression. REM sleep is characterized by selective activation of cholinergic neurons in the basal forebrain and brainstem pedunculopontine tegmental nucleus and is associated with electrochemical activation of the cortex, including limbic areas associated with depressive symptomatology. The serotonergic raphe and noradrenergic locus coeruleus are practically silent during REM. Sleep deprivation, particularly during REM periods consistently improves mood. The cholinergic theory of depression gains added support by a serendipitous finding, which demonstrates the robust and rapid onset antidepressant and anxiolytic effects of the antimuscarinic agent scopolamine (Furey and Drevets, 2006). Many of these patients had chronic depression and failed to respond to conventional treatments.

An indirect measure of cholinergic supersensitivity is the increase in growth hormone response to pyridostigmine in depressed patients (O'Keane et al., 1992). Pyridostigmine is an acetylcholinesterase inhibitor and an indirect muscarinic agonist. Further work on GH (growth hormone) responses is one measure validating the concept of a cholinergic supersensitivity underlying an affective spectrum. Enhanced GH responses to pyridostigmine challenge also occur in panic disorder (Cooney et al., 1997) and obsessive compulsive disorder (OCD) (Lucey et al., 1993) and it was suggested that the cholinergic supersensitivity in OCD could arise as a secondary phenomenon due to serotonergic dysfunction. Limitations of the former study were the considerable variability in GH responses between subjects and the depressive comorbidity. However, in the latter study, a marked GH response was observed in every patient. None were depressed at the time of the study nor had they been on any medication for at least 6 months. Muscarinic cholinergic supersensitivity due to altered serotonergic function is actually proposed to underlie the extensive degree of comorbidity between these disorders. A monoaminergic-muscarinic imbalance would suggest a relative rather than absolute hypercholinergic state.

## Cortisol

The role of cortisol in hippocampal regulation of cortical monoamine levels may unify the spectrum of affective clinical states. One model of non-psychotic depression states that elevated levels will interfere with hippocampal- and amygdala-related phasic release of monoamines and thus, reduced levels of these neurotransmitters will result in the observed breadth of neurocognitive deficits (Vakalopoulos, 2007). In depressed patients a negative correlation was found between salivary cortisol levels and hippocampal related verbal and visuospatial memory performance and executive function (Hinkelmann et al., 2009). Corticosterone-induced decrease in terminal release of serotonin by the reuptake inhibitor fluoxetine was recently shown in rats (Gartside et al., 2003). The reduced response to the SSRI can be explained by the direct effect of corticosterone on the hippocampus and thus a decrease in the phasic activation of the serotonergic midbrain raphe. It can be a factor in modeling drug resistance. A positive correlation was recently demonstrated between remission rates in depression and posterior hippocampal volumes (MacQueen et al., 2008). Another study demonstrates the shorter latency improvement in depressive symptoms when the steroid synthesis inhibitor metyrapone is added (Jahn et al., 2004).

Evidence from the use of corticosteroids in medical therapy implies a role for cortisol in affective disorders. A recent review of the literature concludes that the use of corticosteroids, especially in higher doses, is clearly associated with both mood and cognitive changes (Brown and Chandler, 2001). Dissociable memory impairments have also been documented (Varney et al., 1984). This latter study of six patients referred for the assessment of dementia had reversible prednisone related deficits in verbal and non-verbal explicit recall, attention, concentration and mental speed. These were not attributed to steroid psychosis as four of the patients did not exhibit any such symptoms and in the other two psychotic symptoms had resolved with persisting cognitive impairment. Stress level cortisol treatment impairs verbal memory in normal subjects (Newcomber et al., 1999). The deficits occurred early in the treatment within 4 days. Direct impairment of hippocampal function is an assumed factor.

Chronic effects of stress hormones on hippocampal volume are well documented, however, evidence also exists for acute inhibitory dysregulation of neuronal function by glucocorticoids. Intravenous administration of hydrocortisone, mimicking stress effects, caused a peak decline in the activity of the hippocampus and amygdala by 30–35 min (Lovallo et al., 2010). Cortisol readily crosses the blood-brain barrier. Neuronal hyperpolarization was the putative mechanism. Oral ingestion of hydrocortisone decouples the amygdala from the hippocampus, hypothalamus and locus coeruleus in healthy males (Henckens et al., 2012), consequently impairing forebrain noradrenergic activation (Valentino and Van Bockstaele, 2008).

## Depression

The explicit interpretation of subconscious negative emotional constructs such as sadness or anxiety forms a cornerstone of theory behind cognitive behavior therapy. The subconscious appraisal of cues and concomitant expression of mood states by a parallel cognitive structure is established in proposed simple premotor networks (Vakalopoulos, 2005a,b). The M1-type muscarinic cholinergic receptor plays a key role in laying down such traces and the learning of rigid maladaptive behavior. The importance of this unconscious cognition is that it establishes a habitual way of feeling or interpreting cues within the environment, i.e., a negative cognitive bias so typical of depression. The enduring presence of negative mood states and associated cognitive thinking is mediated by the relative inflexibility of automated networks in general, and due to a genetic vulnerability and/or an acquired pathophysiology resulting in the disruption of mechanisms for the reversal of learning of negative emotional states.

Consistent with a primary hippocampal stress-induced pathophysiology, Sheline et al. (1996) demonstrated reduced hippocampal volumes on MRI for 10 subjects with recurrent major depressive disorder. A large postmortem study of major depressive subjects found a 30–40% increase in packing density and decreased size of neurons in the CA1–CA3 subfields and dentate gyrus (Stockmeier et al., 2004). However, intraday improvements in cognitive performance associated with diurnal variation in cortisol levels (Moffoot et al., 1994) suggest a rapidly reversible component of hippocampal dysfunction that is not exclusively related to atrophy. Hippocampal lesions are associated with deficits in anterograde memory and reversal of learning in both humans and animals (Vakalopoulos, 2006). Vakalopoulos (2007) presents a detailed account of cognitive deficits in major depression. This latter paper explores the findings of psychomotor retardation, poor attention, impulsiveness and deficits in explicit memory as a function of a general reduction in monoamine levels directly brought about by hippocampal dysfunction. Thus, impaired phasic activation of dopaminergic neurons would compromise working memory and overlapping systems of control for motivation and motor performance (Salomone, 1992; Moffoot et al., 1994). That of catecholaminergic neurons in general would affect attention and behavioral inhibition. The proposal that hippocampal dysfunction would hamper phasic serotonergic activation would alter the efficiency of explicit memory acquisition and reversal of learning. The current hypothesis also accounts for the postulated secondary changes in density and size of neurons of the dorsolateral prefrontal cortex, orbitofrontal cortex and anterior cingulate cortex to a reduction in the trophic activity of dopaminergic, noradrenergic, and serotonergic projections (Rajkowska, 2006).

### The medial habenula (MHb) mediates dorsal raphe activation by the hippocampus

**Figure 3:**
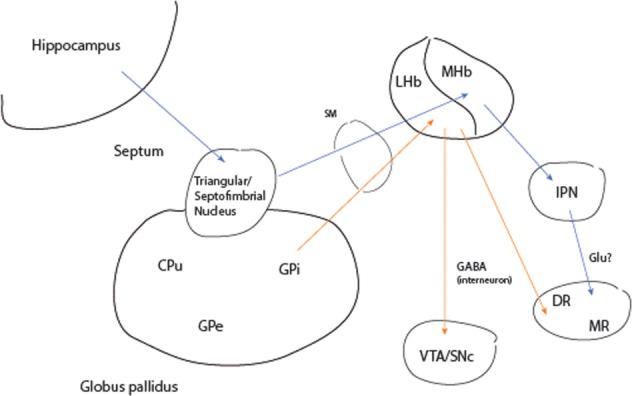
**Activation of monoaminergic nuclei by the hippocampus and globus pallidus**. Blue arrows represent transmission pathway of hippocampal theta to serotonergic raphe. Orange arrows input and output of the lateral habenula. CPu, caudate putamen; DR, MR dorsal and median raphe; GABA, gamma-aminobutyric acid; Glu, glutamate; GPe, GPe, globus· pallidus external and internal segments; IPN, interpeduncular nucleus; LHb, MHb, lateral and medial habenula; SM, stria medullaris; SNc, substantia nigra pars compacta; VTA, ventral tegmental area.

Evidence for a hippocampal pathway to brainstem monoaminergic nuclei comes from a unique case of remission to deep brain stimulation (DBS) in a 64 year-old woman with chronic intermittent depression on maintenance electroconvulsive treatment (ECT) (Sartorius et al., 2010). Bilateral electrode placement was in the stria medullaris, a major afferent pathway to the habenula complex (Figure 3). As an explanation of the treatment mechanism the authors focus solely on the input from the lateral habenula (LHb) to the dorsal raphe (DR) and ventral tegmental (VTA) areas, sources of cortical serotonergic and dopaminergic projections, respectively. The LHb receives afferents from the internal segment of the globus pallidus (GPi) and lateral hypothalamus. However, septal input to the medial habenula (MHb) also traverses the stria medullaris. The MHb reaches the DR indirectly via the interpeduncular (IPN) nucleus and was first proposed as a pathway of phasic activation of this midbrain neuronal cluster by the hippocampal theta rhythm (Vakalopoulos, 2006). Thus, DBS would be expected to mimic activation of the DR by the hippocampus, a structure that is often atrophied or functionally impaired especially in the context of chronic severe depression.

Glutamatergic projections from both the medial prefrontal cortex (mPFC) and LHb inhibit firing of a serotonergic raphe cells through GABAergic interneurons (Varga et al., 2003). However, coextensive lesions of the medial and LHb in rats completely block the rise in DR 5-HT levels in response to fear conditioning (Amat et al., 2001). Basal levels remain unchanged, so the habenula mediates a phasic excitatory input. The studies suggest an alternative structure to the LHb namely, the MHb as providing excitatory input to the serotonergic DR. Lesions of the fasciculus retroflexus (FR) in the neonate rat, the efferent arm of the habenula complex, increase open arm avoidance in rats on an elevated plus-maze, a widely used measure of anxiety (Murphy et al., 1996). Habenula lesions also compromise mnemonic function as illustrated by a more complex or effortful protocol to a one-way active avoidance of shock task (Thornton and Bradbury, 1989). This is due to the MHb as kainic acid lesions of the LHb, which spared MHb neurons, showed normal avoidance responses compared to electrolytic lesions of LHb, which damaged efferent fibres of passage from the MHb (Wilcox et al., 1986). Damage to the IPN also impairs conditioned avoidance, but not retention of the freezing (Thompson, 1960). In the latter study, lesions were made 4 h after criterion performance and retesting 7 days later suggesting a deficit in consolidation and relearning avoidance.

Two-way shuttle-box conditioned active avoidance is significantly enhanced by lesions of the septum, stria medullaris-habenular complex and fornix, but showed resistance to extinction (Van Hoesen et al., 1969). A similar finding was demonstrated for lesions of the IPN, but not the VTA (Wilson et al., 1972). Conversely rats were impaired in a passive avoidance (PA) protocol, which resembles failure of inhibition to search out food reward and parallels impulsive behavior in human depressives. The dissociation in learning conditioned fear responses with septal-MHb-IPN pathway lesions is an important dichotomy, which mirrors manipulations of serotonergic function (Vakalopoulos, 2006). Reduced monoaminergic tone facilitates procedural skills mediated by higher cholinergic tone and parallels resistance to changes in maladaptive behaviors. Increased REM sleep occurs in rats, which reach criterion performance in two-way active avoidance conditioning (Portell-Cortés et al., 1989). REM sleep is associated with high cholinergic activity, but almost quiescent serotonergic and noradrenergic firing rates.

## Gender differences

Gender has a significant effect on the expression of mental illness. For example, women have a later age of onset of schizophrenia and less severe symptoms than men. Female gonadal hormones appear to be protective. Oestrogen and progesterone prevent buspirone and 8-Hydroxy-N, N-dipropyl-2-aminotetralin or 8OH-DPAT (agonists at 5-HT1AR) induced disruptions of prepulse inhibition (PPI) in humans and in rat models (Gogos and van den Buuse, 2004; Gogos et al., 2006). PPI is a measure of sensorimotor gating and is impaired in schizophrenia (Swerdlow et al., 1992). PPI is sensitive to cholinergic muscarinic manipulations. Oestrogen potentiates the effects of the cholinergic agonist arecoline in a T-maze foot shock experiment when injected directly into the hippocampus (Farr et al., 2000) and oestrogen treatment reverses some of the cognitive effects of anticholinergic drugs in post-menopausal women (Dumas et al., 2006). Cholinergically-mediated convergent mechanisms in major depression explain the 2-fold greater number of females than men suffering the disorder. If one assumes that part of the therapeutic effect of tricyclic antidepressants (TCAs) depends on its anticholinergic profile then, the model also explains the relative resistance to TCAs in pre-menopausal as compared to post-menopausal women.

In preliminary studies by O'Keane and Dinan (1992) the GH response to pyridostigmine in the luteal phase was twice that in the follicular phase suggesting again a cholinergic hypersensitivity that correlates with the clinical decrease in mood. The mechanism is likely to be correlated with increases in oestrogen and progesterone in the luteal phase. After menopause the rate of depression in women declines and resembles that of men. In a recent study the chronic administration of oestrogen therapy in post-menopausal women induced marked negative emotional responses to a stressful event (Newhouse et al., 2007). The response is independent of monoamine depletion, which suggests the involvement of other neurotransmitters.

## Cognition and the dysregulation of affect

The paradigm of elevated cortisol related hippocampal damage, as a causal mechanism of reduced neocortical monoaminergic neurotransmitter levels with relative sparing of cholinergic phasic activation and neuromodulation has heuristic significance for a number of allied and frequently comorbid conditions to the affective disorders. As a group they typically respond like major depression to serotonin and noradrenaline reuptake inhibitors suggesting common biological pathways to dysfunction.

### Post-traumatic stress disorder

Post-traumatic stress disorder (PTSD) may result from exposure to extreme stressors such as childhood physical and sexual abuse and military combat. Complaints of cognitive disturbances especially memory and concentration are commonly reported. Objective deficits in declarative memory, verbal recall in particular, have corroborated this impression (Bremner et al., 1993) as have the reduction in hippocampal volume and function during encoding (Bremner et al., 1995, 2003).

Although intrusions are vivid conscious images they are often triggered automatically by contextual cues, both internal and external, that may be described as subconscious. A dual representation theory posits that deliberately retrievable memories are qualitatively distinct from spontaneous reenactments, the former accessed verbally (Brewin et al., 1996) and the latter implicitly by association with unconscious fear conditioning (Elzinga and Bremner, 2002). Contextualized cues may function in a preattentive or alerting sense to focus conscious attention on implicit affective preoccupations. Verbal cues and ruminative thoughts are often described by patients as being on the margin of awareness (Beck, 1976). Evidence for intact or even enhanced non-declarative memory relative to impaired explicit recall comes from a number of sources. Recall following priming by word stems is facilitated for combat related words (Zeitlan and McNally, 1991; Amir et al., 1996). A delay in color-naming for trauma related words is found for both rape victims (Foa et al., 1991) and Vietnam veterans (McNally et al., 1993). Subjects with PTSD show greater initial eye fixations of threat words than controls (Bryant et al., 1995) supporting preattentive or subliminal attraction. Another study actually demonstrated a delayed recall deficit in rape victims with PTSD, but intact implicit memory (Jenkins et al., 1998).

Generalized cognitive deficits have been found in sustained attention and increases in errors of commission, intrusions and general response inhibition independent of comorbid, but analogous to major depression (Vasterling et al., 1998). The resolution of symptoms requires activation of fear networks (Creamer et al., 1992) and assumes M1/5-HT1A receptor plasticity at the synaptic level and impaired if serotonin levels are low as in chronic PTSD. Trauma related amnesia, often termed psychogenic, has its putative origin in the cortisol related impairment of hippocampal theta.

The pathological expression of fear, anger, irritability and impulsiveness results from a failed monoaminergic-mediated cognitive inhibition. This could provide an alternative to the standard explanation of the effects of yohimbine an α2R antagonist. Its administration provokes intrusive memories, flashbacks and high states of arousal (Southwick et al., 1997) and is usually attributed to increased noradrenergic activation. However, blocking adrenergic Gi antagonism of M1R and thus unmasking implicit fear networks is a theory more consistent with the finding that MAO inhibitors such as phenelzine, which also increase noradrenaline levels are an efficacious treatment. By analogy, the SSRI paroxetine would reduce PTSD parameters of reexperiencing, hyperarousal and avoidance (Marshall et al., 2001) by acting on the inhibitory 5-HT1A, Gi receptor. Clonidine an α2 agonist is similarly effective by its post-synaptic antagonism of unconscious fear circuits. In support, slower color naming of trauma words in the stroop task appears to reflect failure to inhibit preattentive processing rather than simply prior priming (McNally, 1998). The prediction of a more general deficit in cognitive inhibition is corroborated by a study of victims in a stroop task where delayed latencies in color naming were present for all words not just trauma words (Cassidy et al., 1992).

### Borderline personality disorder

Borderline personality disorder (BPD) is marked by emotional dysregulation, impulsivity together with instability in interpersonal relationships. It is often attributed to early adversity or abuse and associated with insecure parental attachment, although these factors are neither necessary nor sufficient. Subconsciously motivated outbursts of anger are automatically triggered by the presence of appropriate cues (Meyer et al., 2005). Neurobiological and cognitive features of BPD show similar deficits to those demonstrated in affective disorders. These include perseveration errors in the Wisconsin card sorting test (Lenzenweger et al., 2004) and verbal memory deficits in recall and recognition (Kurtz and Morey, 1999). Borderline patients have up to a 16% reduction in bilateral hippocampal volumes (Driessen et al., 2000). Preliminary evidence for treatment response to SSRIs looks promising (Binks et al., 2006) especially for rapid mood swings (Rinne et al., 2002). A study has implicated ACh in the core symptoms of BPD (Steinberg et al., 1997). Physostigmine, a cholinesterase inhibitor which increases ACh levels in the synaptic cleft, significantly increased both depressive and anger symptoms.

The SSRI fluvoxamine reduces responsiveness of the HPA axis in female BPD patients (Rinne et al., 2003). This occurs presumably and at least in part, by dampening cholinergic hypersensitivity and is based on findings that cholinomimetics elevate multiple pharmacological parameters of the HPA system (see review by Janowsky and Overstreet, 2000). Also, at least in the case of dopamine, brain monoamine depletion causes a supersensitive adrenocorticotrophic hormone (ACTH) response to physostigmine in rats (Downs et al., 1986).

### Obsessive-compulsive disorder

OCD presents clinically with repetitive intrusive thoughts and stereotyped compulsive behaviors that are distressing to the individual and interfere with normal functioning. It is considered an anxiety disorder and there are a number of clinical features that place OCD in the broader paradigmatic spectrum of a failure of cognitive inhibition. Indeed there is a high comorbidity of OCD with other psychiatric disorders. Patients suffer from impulsiveness and impaired response inhibition (Aycicegi et al., 2003; Ettelt et al., 2007). Neurocognitive deficits are present including prefrontal dysfunction and deficits in spatial working memory and visual and verbal delayed recall (Purcell et al., 1998; Shin et al., 2004; Roth et al., 2005). Motor retardation, as expressed by prolonged movement times and execution have also been demonstrated (Purcell et al., 1998). As have distraction by competing stimuli and perseveration in an attention set-shifting task (Veale et al., 1996). Thus, hippocampal dependent processes are impaired and prepotent behaviors form the core symptoms of OCD. A study showed that OCD is associated with significant hippocampal volume reduction and increased amygdala size (Kwon et al., 2003).

The recommended pharmacological treatment for OCD is a SSRIs like clomipramine or the newer more selective agents. Behavior therapy, as in major depressive illness can reduce likelihood of relapse upon discontinuation of drugs (McDonough and Kennedy, 2002). Consistent with the hypothesis of an antagonistic effect of 5-HT on muscarinic signaling comes from case studies of atypical antipsychotic use. Clozapine and risperidone used in high doses in schizophrenia have been known to exacerbate or induce OCD symptoms *de novo* (Patil, 1992; Kopala and Honer, 1994). Risperidone has antiserotonergic properties and clozapine a muscarinic agonist profile of its secondary metabolite. In a case of SRI resistant OCD, symptoms worsened with the use of risperidone, but markedly resolved with olanzapine augmentation of fluoxetine (Potenza et al., 1998). Olanzapine has antimuscarinic effects and less of a procholinergic profile than clozapine. That acetylcholine plays an important role in the pathophysiology of OCD has been suggested (Carlsson, 2001). This is based on the observation of a dense cholinergic innervation of the amygdala and the effectiveness of clomipramine in treatment, which has anticholinergic properties.

### Other anxiety disorders

Panic disorder, generalized anxiety disorder, social phobia are part of a group of anxiety disorders that have generally shown inconsistent results on neuropsychological tests from the little empirical data that exist. Several studies do show deficits in verbal and non-verbal episodic memory (Lucas et al., 1991; Asmundson et al., 1995) and executive function (Cohen et al., 1996). A recent study has replicated the findings for verbal memory and executive function in a trail making task (Airaksinen et al., 2005). Decreased left temporal volume has been reported in panic disorder patients with a trend toward smaller left hippocampal volume (Uchida et al., 2003). That hypothalamic pituitary adrenal (HPA) axis dysfunction may underlie the findings is suggested by exaggerated cortisol and ACTH response to novelty (Abelson et al., 2006). In fact, dexamethasone suppression test (DST) non-suppression in patients treated for panic disorder predicts a poorer prognosis at 3 year follow up (Coryell et al., 1991) and elevated 24 h cortisol levels is associated with continuing disability 2 years later (Abelson and Curtis, 1996). Anxiety disorders generally respond well to SSRIs such as fluvoxamine (Asnis et al., 2001). A recent study of patients with social anxiety disorder, of whom half had comorbid agoraphobia demonstrated up to a 30% reduction in binding of the 5-HT1AR in limbic regions including the insula, amygdala, and anterior cingulate (Lanzenberger et al., 2007). It is not known whether this is genetically determined or an acquired dysfunction. Cholinergic mechanisms are thought to play a major role in panic disorder and the use of the muscarinic antagonist biperidin reduces the subjective anxiety of panic disorder patients to CO2 inhalation (Battaglia et al., 2001).

## Genetic and animal studies

The role of serotonin in both declarative memory and adaptation is supported by a number of recent studies. Human carriers of the rare allele of 5-HT2A receptor, associated with blunted receptor response, have up to 20% poorer performance on two different declarative memory tasks (De Quervain et al., 2003). Prefrontal serotonin depletion in the marmoset leads to cognitive inflexibility in a serial discrimination reversal task (Clarke et al., 2004). Fear extinction in mice is accelerated by blocking PKC (protein kinase C) and not affected by PKA, protein kinase A (Tronson et al., 2008) consistent the involvement of Gi-protein complex and 5-HT1A-M1 interactions in reversal learning.

The Porsolt swim test, which is a model of learned helplessness has been used as an indicator of behavioral depression. The injection into the rat nucleus accumbens of the muscarinic agonist arecoline increased while the M1 antagonist pirenzipine decreased behavioral depression (Chau et al., 1999). Likewise fluoxetine and the 5-HT1A agonist 8-OH-DPAT increase swimming time analogous to the effect of pirenzipine suggesting antagonism of M1 receptors. M1 knockout mice mimic reduced immobilization in the Porsolt forced swim test (Miyakawa et al., 2001). The muscarinic M1 receptor is necessary for PA learning, an aversive learning test (Gherlardini et al., 1999). The muscarinic agonist oxotremorine strongly enhances contextual memory in a fear conditioning paradigm (Vazdarjanova and McGaugh, 1999) and inhibitory avoidance as measured by retention latency (Introini-Collision et al., 1996) by modulating amygdala function. Importantly, propranolol does not attenuate the effects of oxotremorine. The data are consistent with an implicit system of fear conditioning, facilitated by M1 muscarinic receptor and is in addition to the amygdala-dependent declarative enhancement of emotional memory mediated by post-synaptic adrenergic receptors (Cahill and McGaugh, 1998). Further evidence comes from α-adrenergic receptor effects on inhibitory avoidance (Feery et al., 1999). Phenylephrine, a non-specific α-adrenoceptor agonist tended to impair retention when injected into the BLA. When given concurrently with the α2 antagonist yohimbine, phenylephrine significantly improved retention latencies to enter the dark compartment. The α1 antagonist prazosin also significantly impairs retention. Thus, whereas the α1-adrenoceptor enhances the α2-adrenoceptor impairs memory, which is accounted for by the inhibition of M1 muscarinic-mediated mechanism.

5-HT1A receptor antagonists and the acetylcholinesterase inhibitor physostigmine, which increases synaptic ACh levels, facilitate increased latencies in entering the dark compartment associated with footshock, while the 5-HT1A agonist 8-OH-DPAT markedly decreases this measure (Madjid et al., 2006). The 5-HT1A antagonist WAY 100635 enhances performances on an object recognition task (Pitsikas et al., 2003), which can be solved implicitly i.e., using familiarity based judgments that are hippocampal-independent. Consistent with M1-5-HT1A receptor interactions in the modification of behaviors this same study revealed a failure of extinction of recognition memory as compared to control rats.

8-OH-DPAT caused impaired PA when injected just prior to a retention test, i.e., after presumably normal acquisition (Misane et al., 1998). This result resembles cognitive inhibition, in this case suppression of the tendency to delay entry into the aversively conditioned compartment, possibly related to a reduced fear response. Whereas 5-HT1A antagonists reverse deficits caused by scopolamine on PA and object recognition tasks, they are generally ineffective in the water maze test (Pitsikas et al., 2003; Luttgen et al., 2005). These findings are consistent with the two-process model where scopolamine inhibits M1 muscarinic receptors and putative implicit processes involved in object recognition and PA and can be reversed by augmenting simple premotor network behavior using 5-HT1A receptor antagonists.

## Toward a scientific psychology

Neurocognitive deficits are rather modest or insignificant in many patients with mild to moderate panic disorder (Gladsjo et al., 1998) and clinical depression (Grant et al., 2001) and hippocampal atrophy is by no means a universal finding. Subconscious learning of negative habits can be resilient to reversal and genetic factors may contribute to persistence of dysfunction. Risk is expressed through certain endophenotypes or personality traits. A possible example of genetic predisposition is met by the finding of allelic variation in 5-HT transporter promoter regions with the short allele variant conferring increasing vulnerability to stressful events (Caspi et al., 2003). Blunted prolactin response to fenfluramine or to the 5-HT2C agonist m-CPP is inversely correlated to aggressiveness (Coccaro et al., 1997). Impaired signal transduction of 5-HT2A platelet receptors in major depression (McBride et al., 1994) also implicates serotonergic dysfunction.

ADHD represents a risk factor not just for depression, but also for BPD (Fossati et al., 2002). Guanfacine, an α2AR agonist enhances general prefrontal function in ADHD including behavioral inhibition (Arnsten, 2006). The receptor acts to inhibit cyclic AMP consistent with Gi-mediated antagonism of prepotent behaviors. One might then expect an overlap with other disorders. Indeed, in one sample of children with OCD under 12 years of age, 57% had ADHD and the younger age group also predicted an increased risk for other anxiety disorders (Geller et al., 2001).

### Targeting the subconscious scaffolding of emotional dysregulation

How does one reconcile the proposed pharmacological mechanism of unconscious remodeling with the clinical efficacy of psychotherapy? The interactions between conscious and unconscious processes have a long theoretical and more recently, empirical tradition. A clue to this most fundamental of relationships is provided by the observation of automatic thoughts in both depressive and anxiety patients (Beck, 1976). Beck describes these as almost reflex, negative thoughts, arising without any prior reflection and appear rather plausible to the subject. Many patients are not even fully aware of these thoughts, although they are generally accessible to consciousness. They are often noticed only after having been trained to direct attention to them before they explicitly recognize their existence and the effects these are having on their emotional states. The question remains do they represent legitimate unconscious structures. A comparison can be drawn here with the experience one may have while reading a text from a book or article where on occasions one is distracted by unrelated reveries. One comes to the realization of having read the preceding paragraph without explicit awareness of content. A subject is trained to pay attention to automatic thoughts generated by the subconscious. Even with spontaneous conscious access to these thoughts, as is often the case with rumination, their content and underlying motivation are so compelling that they form a belief system that is analogous to Beck's schemas. Empirical evidence of a general application of intuitive knowledge without awareness of its principles and in defiance of rational judgment has been repeatedly demonstrated (Epstein, 1994). Fixed, prepotent beliefs were not likely to be irrational in an evolutionary sense, and are an important basis to intuitive skill. However, in an increasingly complex society their relative inflexibility, particularly in vulnerable individuals, may be maladaptive.

## Conclusion

A hopeful outcome of the current hypothesis is a heuristic for future investigation of psychotropics that can aid the treatment of increasingly endemic disorders of significant morbidity and mortality. This is based on a detailed model of understood and proposed neuropharmacological interactions that involve critical neuromodulator systems known to be involved. For example, further research into and modifications to an older generation of drugs called psychotomimetics may prove useful. Low doses of lysergic acid diethylamide (LSD), a potent 5-HT2A/2C agonist, may have some positive mnemonic effects for the specific type of amnesia in patients with focal hippocampal damage. The literature provides some anecdotal instances of psychotomimetic efficacy in the treatment of mood disorder and in particular OCD (Zghoul and Blier, 2003; Nichols, 2004). Most of these drugs have potent 5-HT1A/2A agonist activity. The non-hallucinogenic ergoline lisuride, an analog of LSD, has a profile that could be selectively useful in the treatment of mood disorders.

### Conflict of interest statement

The author declares that the research was conducted in the absence of any commercial or financial relationships that could be construed as a potential conflict of interest.
